# Conditional gene expression in the mouse using a *Sleeping Beauty *gene-trap transposon

**DOI:** 10.1186/1472-6750-6-30

**Published:** 2006-06-26

**Authors:** Aron M Geurts, Andrew Wilber, Corey M Carlson, Paul D Lobitz, Karl J Clark, Perry B Hackett, R Scott McIvor, David A Largaespada

**Affiliations:** 1 Department of Genetics, Cell Biology, and Development and The Arnold and Mabel Beckman Center for Transposon Research at the University of Minnesota, Minneapolis, MN 55455, USA; 2 Institute of Human Genetics, University of Minnesota, Minneapolis, MN 55455, USA; 3 University of Minnesota Cancer Center, Minneapolis, MN 55455, USA

## Abstract

**Background:**

Insertional mutagenesis techniques with transposable elements have been popular among geneticists studying model organisms from *E. coli *to *Drosophila *and, more recently, the mouse. One such element is the *Sleeping Beauty *(SB) transposon that has been shown in several studies to be an effective insertional mutagen in the mouse germline. SB transposon vector studies have employed different functional elements and reporter molecules to disrupt and report the expression of endogenous mouse genes. We sought to generate a transposon system that would be capable of reporting the expression pattern of a mouse gene while allowing for conditional expression of a gene of interest in a tissue- or temporal-specific pattern.

**Results:**

Here we report the systematic development and testing of a transposon-based gene-trap system incorporating the doxycycline-repressible Tet-Off (tTA) system that is capable of activating the expression of genes under control of a Tet response element (TRE) promoter. We demonstrate that the gene trap system is fully functional *in vitro *by introducing the "gene-trap tTA" vector into human cells by transposition and identifying clones that activate expression of a TRE-luciferase transgene in a doxycycline-dependent manner. In transgenic mice, we mobilize gene-trap tTA vectors, discover parameters that can affect germline mobilization rates, and identify candidate gene insertions to demonstrate the *in vivo *functionality of the vector system. We further demonstrate that the gene-trap can act as a reporter of endogenous gene expression and it can be coupled with bioluminescent imaging to identify genes with tissue-specific expression patterns.

**Conclusion:**

Akin to the GAL4/UAS system used in the fly, we have made progress developing a tool for mutating and revealing the expression of mouse genes by generating the tTA transactivator in the presence of a secondary TRE-regulated reporter molecule. A vector like the gene-trap tTA could provide a means for both annotating mouse genes and creating a resource of mice that express a regulable transcription factor in temporally- and tissue-specific patterns for conditional gene expression studies. These mice would be a valuable resource to the mouse genetics community for purpose of dissecting mammalian gene function.

## Background

Derived from ancient salmonid fish sequences [1], the *Sleeping Beauty *(SB) transposon is a member of the Tc1/*mariner *superfamily of cut-and-paste transposable elements [2] and has been developed as a vertebrate transformation tool [3] and germline insertional mutagen [4,5]. We and others have shown that the SB transposon is highly active in the mouse germline and can generate heritable loss-of-function mutations that lead to detectable phenotypes [4-7].

Data accumulated from spontaneous and engineered mouse mutations suggests that a significant percentage of mouse genes are essential for early development. This has necessitated the creation of various genetic tools for conditional loss- or gain-of-function genetic studies. In *Drosophila*, the ability to regulate genes in a tissue- or temporally-specific manner has become one method to study the function of these genes. Likewise, the ability to control the expression of an essential mouse gene is one way to discover its function in tissues that are formed after lethal phenotypes are manifest. Temporal and spatial control of gene expression in mutant backgrounds can be used to determine when a gene product is required during a developmental process [8-10]. With this in mind, and taking lessons from the *Drosophila *literature on GAL4/UAS-based *P*-element transposon systems [11], we designed a SB transposon-based gene trap system that allows us to annotate the expression pattern and function of mouse genes.

Previous studies using the SB transposon system for generating insertional mutations in the germline of mice used various gene- and polyA-trap transposon vectors [4,5]. These vectors were designed to truncate an endogenous mRNA, incorporate coding sequences for a visible reporter like *β*-Galactosidase or green fluorescent protein (GFP), and report the expression patterns of trapped genes. If a transcriptional transactivator, similar to GAL4, were used in place of a visible reporter molecule, a small amount of the molecule could lead to high levels of a secondary reporter transgene under control of a responsive promoter. Analogous to the GAL4/UAS system, the tetracycline-controlled transactivator (tTA) and Tet-response element promoter (TRE) were developed for mammalian systems [12].

Here we report the creation of a mutagenic SB-based, gene-trap transposon vector that can insert into a mouse gene and express the Tet-off transcriptional transactivator (tTA) in its tissue- and temporally-regulated manner. The "gene-trap tTA" can mutate genes and record their expression patterns for functional annotation as well as create a mechanism for driving the expression of transgenes in a regulable manner *in vivo*. We also report several general parameters that impact the application of the *Sleeping Beauty *transposon system to mouse functional genomics including analysis of transposon mobilization rates in several different transgenic lines and the rescue of a mutant phenotype by remobilization.

## Results

### Creation of the gene-trap tTA system

**Figure 1:**
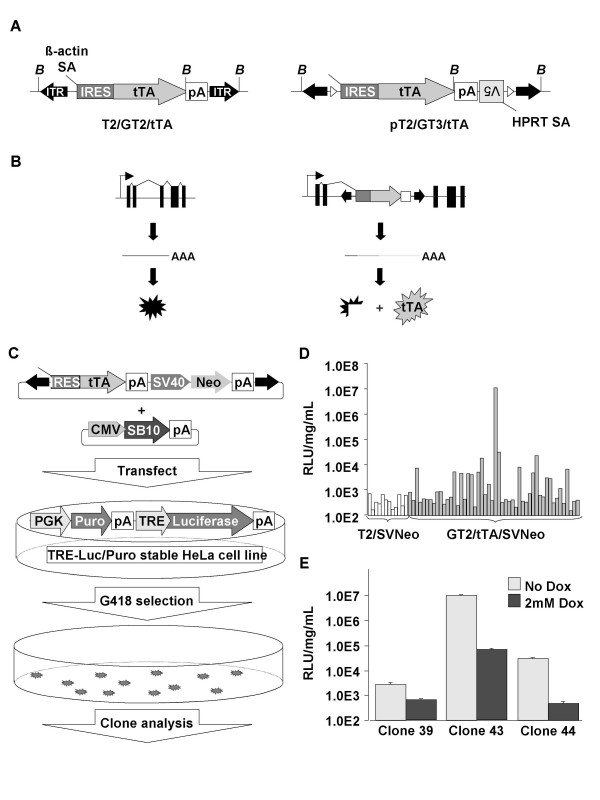
**Gene-trap tTA vector design and *in vitro *testing A)**. The T2/GT2/tTA and T2/GT3/tTA vectors. The 'GT2' version is capable of mutating genes in one orientation while the 'GT3' version can mutate genes in both orientations upon insertion into a gene. (***B***- *Bam*HI sites **) B) **Compared to normal expression (left), when the T2/GT2/tTA SB transposon-based gene-trap vector inserts into a gene in the direction of transcription (right), endogenous splicing incorporates the IRES and tTA sequences and the bicistronic mRNA is prematurely truncated at the SV40 late polyadenylation site. The bicistronic mRNA allows cap-independent translation of the tTA molecule in addition to any peptide encoded by upstream exons. **C) **A stable, TRE-regulated luciferase cell line was created to test the T2/GT2/tTA/SVNeo transposon vector. After co-transfection with a plasmid source of transposase, G418^R ^clones were individually expanded for analysis. **D) **Individual luciferase expression levels of G418^R ^clone cell extracts from twelve control (pT2/SVNeo) and fifty gene-trap tTA clones from **C**. **E) **Incubation of three clones from **C **in media supplemented with 2 mM doxycycline results in 10- to 100-fold reduction of luciferase expression.

A SB transposon-based gene-trap tTA vector was first designed for and tested in zebrafish embryos [13]. The gene-trap tTA vector was shown function in somatic tissues of zebrafish and activate a TRE-regulated GFP transgene. T2/GT2/tTA and T2/GT3/tTA (Fig. 1A) are similar to the earlier gene-trap tTA (GT/tTA) and encode a carp β-actin splice acceptor, the encephalomyocarditis virus (EMCV) internal ribosomal entry site (IRES), the Tet-Off^® ^(tTA) coding sequence (Clontech, Palo Alto, CA), and the SV40 late polyadenylation signal. Modifications are described in the Materials and Methods and include an upgrade to the 'T2' versions of the SB inverted terminal repeat sequences (ITR), demonstrated to increase transposition activity *in vitro *[14], and the addition of stop codon sequences in each reading frame just upstream of the IRES. The T2/GT3/tTA transposon vector (Fig. 1A) was additionally modified to include a splice acceptor from the mouse hypoxanthine phosphoribosyltransferase gene (HPRT) and a V5-epitope tag on the opposite strand of the transposon. This allows the gene trap to disrupt gene expression upon insertion into a mouse gene in either orientation. If open reading frame one were conserved after splicing from the endogenous exon, the V5-epitope would be added to the truncated peptide.

Fig. 1B shows the method by which the gene-trap tTA can disrupt a mouse gene. When the gene-trap transposon lands in the intron of a gene, it will intercept splicing from an upstream exon and incorporate the IRES and tTA elements to form a bicistronic messenger RNA. Translation of the endogenous gene peptide is truncated at one of the stop codons incorporated just upstream of the IRES. The IRES element allows cap-independent translation initiation from the second open reading frame to produce tTA from the same mRNA. For the T2/GT2/tTA vector, an insertion into a gene in the opposite orientation is not predicted to mutate the gene, however, the T2/GT3/tTA would interrupt splicing with the HPRT splice acceptor, but will not express tTA.

Before making transgenic mice, we validated the functionality of the gene-trap tTA as a faithful activator of reporter gene expression *in vitro*. First, a G418-resistance cassette was cloned into the T2/GT2/tTA transposon vector to generate T2/GT2/tTA/SVNeo (Fig. 1C). We then generated a HeLa cell line harboring a TRE-regulated luciferase transgene. Upon co-transfection of the pT2/GT2/tTA/SVNeo transposon vector plasmid and a source of SB transposase, pCMV-SB [1], the gene-trap tTA transposes at random into the HeLa cell genome. The SVNeo cassette confers G418 resistance to cells that integrate and express the transposon vector, but the gene-trap tTA cassette can intercept splicing and express tTA only if the transposon lands in an expressed gene in the correct orientation. Fig. 1D shows the luciferase activation in sixty-two individual G418-resistant clone extracts. As a control, a transposon that contained the same G418 resistance cassette, but not the splice acceptor-IRES-tTA cassette, did not activate luciferase expression (Fig. 1D, white bars). As expected, only a subset of T2/GT2/tTA/SVNeo-transgenic clones, 17 out of 50, showed activation of TRE-luciferase above background levels.

The activity of tTA is regulable by administering the drug tetracycline or its analog doxycycline [15]. We tested this sensitivity by administering doxycycline to three of the TRE-luciferase activated clones from Fig. 1D. Fig. 1E shows the doxycycline-dependent repression of TRE-luciferase activation in each clone. Luciferase activity was reduced approximately two orders of magnitude in clones, 43 and 44, and about one order of magnitude in clone 39. Finally, to assure that gene trap tTA activation was dependent on splicing from an upstream exon of an expressed gene, 5' rapid amplification of cDNA ends (RACE) was performed with primers specific to the gene trap on mRNA extracts prepared from several clones. Additional file 1 shows the sequence tags, chromosomes and gene identification obtained for seven clones. In each case, an upstream exon of the gene is spliced into the gene-trap tTA at the predicted position (underlined). These data demonstrate that the tTA molecule can be expressed from the gene-trap tTA vector in the context of a trapped gene to activate a TRE-regulated transgene and that this activation is repressible by the addition of doxycycline. With the prospect of generating mouse strains with regulated gene-trap tTA activation, we set out to test the system *in vivo*.

### Parameters affecting germline mobilization rates

Transgenic mice were generated with the T2/GT2/tTA and T2/GT3/tTA transposon vectors by standard pronuclear injection (see Materials and Methods). A typical result of this protocol is the head-to-tail concatenation of transgene units before integrating into the mouse genome to create a multi-copy array of transgenes in a mouse chromosome. Each transposon transgene concatemer, thus, serves as a donor site containing multiple substrates for mobilization by the SB transposase. To determine the number of gene-trap tTA transposons in each transgenic line, we performed Southern blot analysis using a restriction digest scheme that resolves the concatemerized transposons to a single band and estimated the number of copies based on its intensity (see Materials and Methods). Table 1 shows ten independent transgenic founder lines with transposon copy numbers ranging from approximately three to sixty copies. To test for mobilization of the transposons in the germline, each transgenic line was crossed to the CAGGS-SB10 transposase-expressing strain [16]. The resulting doubly transgenic "seed" mice were outcrossed to wild type animals to observe gene trap mobilization in the germline. Southern blot analysis on tail biopsy DNA from offspring of seed mice was used, as previously described [4,16], to observe the number of new transposon insertions per gamete. We averaged the insertion data from seed mice from each line to calculate the likelihood (rate per copy number) that a transposon could be mobilized from their respective concatemers (Table 1). The data demonstrates that lines with fewer copies of the transposon substrate show the lowest mobilization rates. This, however, is not a strict correlation. This is evident in comparing lines 6632 and 6657, where the former has fewer copies, but shows a higher per-copy-transposon mobilization rate of the latter.

**Figure 2:**
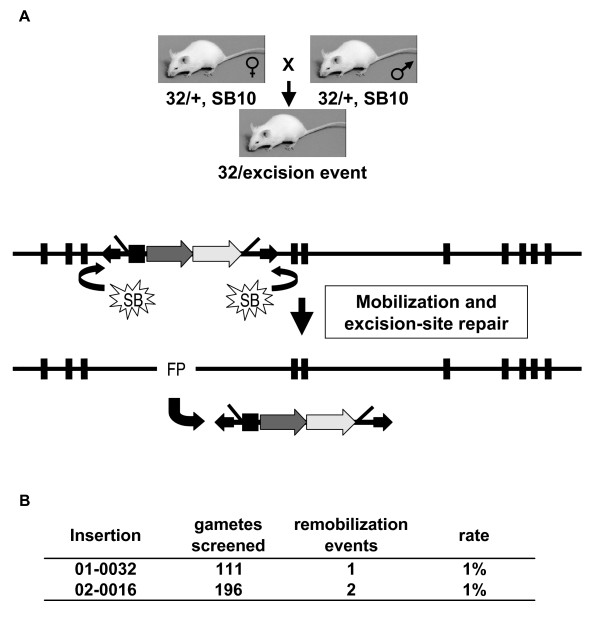
**Phenotypic rescue by transposon mobilization A)**. Breeding scheme to remobilize a transposon insertion, 01-0032, out of the mouse *Slc25a22 *gene as previously reported by Carlson, *et al *. (2003). Animals for both the single-copy transposon insertion 01-0032 and the CAGGS-SB10 transposase were intercrossed. **B) **Germline single-copy remobilization rates of the independently generated insertions 01-0032 and 02A-0016 (Additional file 2) were detected by analyzing the donor site for evidence of a transposon footprint by sequencing (data not shown).

The inability to mobilize transposons in transgenic mice with few transposons in the concatemer is consistent with our efforts to re-mobilize a single copy insertion in the genome. We analyzed the mobilization of two independently generated single copy insertions. Insertion 01-0032 was generated and described in an earlier report [4] and 02A-0016 is a gene-trap tTA insertion identified in an offspring of a T2/GT2/tTA seed male (line 4583) as described below. When SB transposons are excised from a chromosome locus during gametogenesis, host DNA repair machinery typically creates a "footprint" of a few or several base pairs while repairing the double-strand-break gap [17]. We mobilized the insertions 01-0032 and 02A-0016 by intercrossing doubly transgenic mice that harbor a single-copy transposon insertion and the CAGGS-SB10 transposase transgene (Fig. 2A). We detected transposition in about one out of every 100 offspring for either insertion by PCR amplifying the sequences that flank the donor site and sequencing to detect a footprint (Fig. 2B). Notably, remobilization of insertion 01-0032, which causes an early recessive embryonic lethal phenotype as a result of disruption of the mouse *Slc25a22 *gene [4], restores activity of the gene and rescues the recessive lethality associated with this insertion as heterozygous 01-0032/footprint mice are phenotypically normal mice. The inability to efficiently remobilize a single copy insertion has been reported elsewhere [6]. For this reason, we incorporated loxP sites into the T2/GT3/tTA vector (Fig. 1A), flanking the mutagenic portion of the gene-trap vector. Cre-mediated recombination in tissues and in the germline have been demonstrated and are significantly higher than the 1% mobilization rate we can achieve by remobilizing single copy insertions [18-20]. Thus, a T2/GT3/tTA gene trap disruption of a mouse gene might be rescued by expression of Cre recombinase in a tissue- or germline-specific manner.

Table 1 also demonstrates that germline mobilization rates are lower in the female germline when compared to their male siblings (dashed boxes). Finally, the solid box shows the difference in transposition rates between two sibling male seed mice, one hemizygous and one homozygous for the transposase transgene. While the SB10+/+ seed male did not show increased transposition over other independently generated seed mice for the 4563 line, there was an increase over its sibling SB10+/- seed male, suggesting that we may be able to increase transposition rates by increasing the transposase dosage in future studies. The analysis of several transposon-transgenic lines has thus revealed parameters that affect transposition rates, and suggest careful line selection will be required for mutagenic programs.

### *Sleeping Beauty*-mediated gene insertion *in vivo*

**Figure 3:**
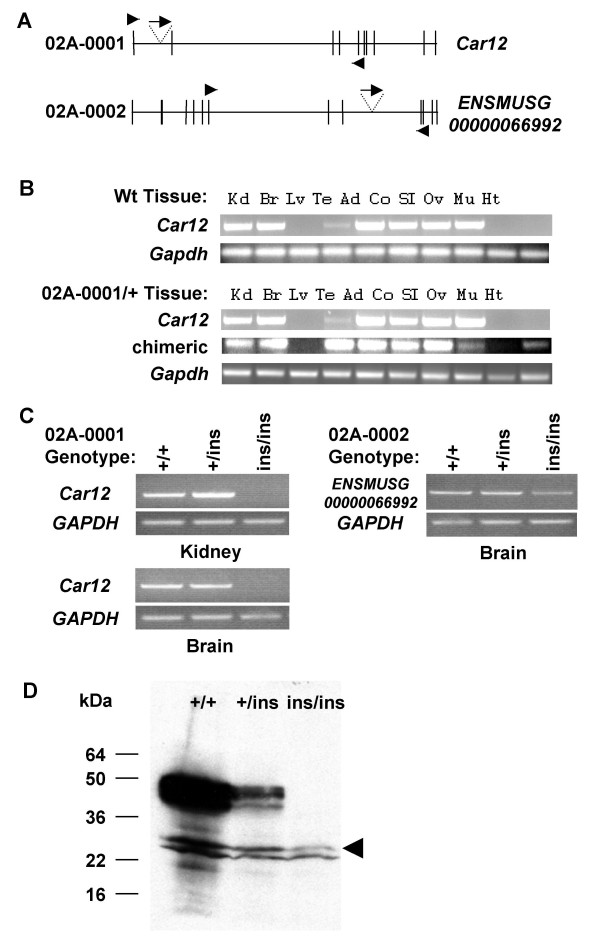
**Molecular characterization of gene-trap tTAfunction A)**. Insertions 02A-0001 in the mouse *Car12 *gene and insertion 02A-0002 in the mouse *ENSMUSG00000066992 *gene. Genes are shown in the orientation of transcription from left to right, vertical lines are exons, along with the position and orientation of the T2/GT2/tTA insertion (arrows). **B) **RT-PCR detection of *Car12 *(top) and *Car12*-IRES-tTA chimeric (bottom) transcription in wild type and 02A-0001 carrier tissues. **Kd**-kidney, **Br**-brain, **Lv**-liver, **Te**-testes, **Ad**-adipose, **Co**-colon,**SI**-small intestine, **Ov**-ovary, **Mu**-muscle, **Ht**-heart **C) **RT-PCR analysis of gene disruption in tissues from wild type (+/+), heterozygous (+/ins), and homozygous (ins/ins) carriers of insertions 02A-0001 and 02A-0002. **C) **Western analysis of CA-XII expression in a wild type, heterozygous and homozygous carrier of insertion 02A-0001. Cross-reaction of the CA-XII antibody with other isoforms serves as a loading control (arrowhead).

T2/GT2/tTA line 4563 and T2/GT3/tTA line 6660 were useful to identify new transposon insertions in the offspring of seed mice. Techniques for cloning transposon-genomic DNA junction sequences have been described [1,4,21]. The chromosomal positions of thirty transposition events in offspring of seed mice from line 4563 were identified by querying the ENSEMBL online mouse genome database using the BLAST function. The T2/GT3/tTA line 6660 was used in a study of mutations induced specifically on mouse chromosome 11 and the insertion data from this line will be reported elsewhere (A.M.G., manuscript in preparation). Additional file 2 and additional file 3 show the data accumulated from the thirty T2/GT2/tTA events. Consistent with previous reports, the T2/GT2/tTA transposons exhibit local hopping, where a significant percentage of new insertions cluster in a particular region of the genome, and the insertions can land in genes [4,5]. Three-primer PCR [3] can readily be used to genotype carriers for any specific transposon insertion. Because we were interested in testing the function of the gene-trap tTA vector in the context of an endogenous mouse gene, carriers of insertions 02A-0001 and 02A-0002, both in mouse genes, (Fig. 3A) were propagated for further study.

### Molecular characterization of gene-trap tTA function

Insertion number 02A-0001 is in the first intron of the carbonic anhydrase-12 gene (*Car12*), while 02A-0002 is inserted into intron 8 of the predicted novel gene *ENSMUSG00000066992 *(Fig. 3A). The top panel in Fig. 3B shows the expression pattern of mouse *Car12 *by reverse-transcriptase PCR (RT-PCR) on RNA extracts from several wild type tissues using primers in exons 1 and 5. If the gene trap is splicing properly, the IRES and tTA sequences should be fused to exon 1 of the *Car12 *mRNA. The second panel shows RT-PCR detection of endogenous *Car12 *and *Car12*-IRES-tTA bicistronic transcripts in RNA samples taken from a heterozygous carrier of insertion 02A-0001. Nested PCR was necessary to detect the fusion transcript in some tissues. Comparing the wild type expression pattern of *Car12 *mRNA and the fusion transcript, it is apparent that expression of the trapped mRNA is tissue specific as predicted. The fusion transcript can be detected in the heart of the carrier, but not wildtype heart RNA extracts. We attribute this to the extra sensitivity of the nested PCR and suspect that endogenous *Car12 *is expressed in the heart at a low level.

If the carp β-actin splice acceptor is efficient, it should truncate the endogenous gene mRNA after the nearest upstream exon. Total RNA extracts from wild type, and heterozygous or homozygous carriers of both insertions 02A-0001 and 02A-0002 were probed for expression of endogenous Car12 or ENSMUSG00000066992 transcripts respectively. Fig. 3C shows complete ablation of Car12 mRNA in the kidney and brain of a homozygous carrier of insertion 02A-0001. Expression of ENSMUSG00000066992, detectable by RT-PCR exclusively in mouse brain (data not shown), was not ablated in homozygous carriers of insertion 02-0002, however, suggesting that the carp β-actin splice acceptor does not work efficiently in each case.

Interestingly, *Car12 *mutant mice demonstrate reduced fitness, as an intercross of 02A-0001 carriers results in non-mendelian inheritance of the homozygous class (data not shown). The expression of the carbonic anhydrase (CA-XII) has been previously localized to the membranes of mouse kidney, colon, and testes [22]. The CA-XII peptide has been previously reported to be 46 kDA [23]. Fig. 3C shows the reduction of CA-XII expression in the heterozygous kidney and absence in the homozygous kidney. Antibody cross-reaction with a ~ 30-kDa peptide is consistent with cross-reactions previously seen in kidney extracts with this antibody [23], and serves as a loading control (arrowhead, Fig. 3C).

### Tissue-specific activation of a TRE promoter-regulated transgene *in vivo*

**Figure 4:**
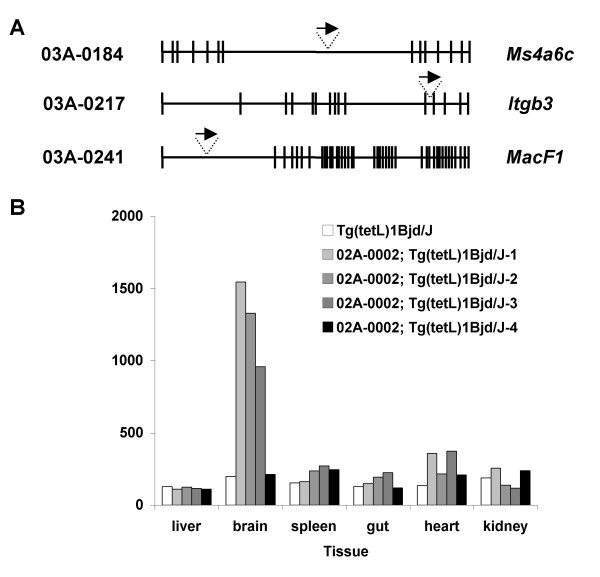
**T2/GT3/tTA gene disruptions and tissue-specific activation of a tet-responsive transgene *in vivo *A)**. T2/GT3/tTA insertions into mouse genes not linked to the original donor site. The genes are shown in the orientation of transcription with vertical lines as exons (from left to right) with the position and orientation of the IRES-tTA trapping cassette (arrow). **B) **Luciferase assays performed on individual tissues from four independent 02A-0002; Tg(tetL)1Bjd/J doubly transgenic and control Tg(tetL)1Bjd/J mice.

The final *in vivo *test for the gene-trap tTA system is to determine whether the vectors can activate a TRE promoter-regulated transgene in a tissue-specific manner. We obtained the Tg(tetL)1Bjd/J strain of mice, transgenic for a TRE-regulated luciferase transgene, from Jackson Laboratories. These mice have been previously shown to respond to tissue-specific tTA expression in a doxycycline-dependent manner [24]. Luciferase activity assays from individual organ extracts from a 02A-0001; Tg(tetL)1Bjd/J doubly transgenic animals showed activation of luciferase in all tissues tested when compared to singly transgenic controls (data not shown). This result was surprising because the trapped gene, *Car12*, is not expressed in the liver (among other tissues) by RT-PCR (Fig. 3B). Insertion 02A-0001 is the result of local transposition, and thus is linked to the original concatemer of transposons. We reasoned that these linked T2/GT2/tTA transgenes present in carriers of this insertion might express tTA, therefore resulting ubiquitous luciferase expression when crossed to the Tg(tetL)1Bjd/J strain. To circumvent this issue, we focused on insertions that were not the result of local hopping and would segregate from other transposable elements. Insertions 02A-0002, 03A-0184, 03A-0217, and 03A-0241 (Figs. 3A and 4A) are the result of non-local transposition of T2/GT2/tTA or T2/GT3/tTA transposons in their respective transposon transgenic strains. Carriers of each of these gene insertions were crossed to the Tg(tetL)1Bjd/J strain of mice with the hope of visualizing tissue-specific, tTA-dependent transactivation of luciferase. As predicted, Fig. 4B shows weak activation of luciferase in the brain of a 02A-0002; Tg(tetL)1Bjd/J doubly transgenic mice, but not other tissues. This pattern was reproduced in three out of four mice. As shown above, this insertion does not completely disrupt the brain expression of *ENSMUSG00000066992 *(Fig. 3C), but some splicing into the T2/GT2/tTA splice acceptor must occur to allow tissue-specific expression of the tTA.

**Figure 5:**
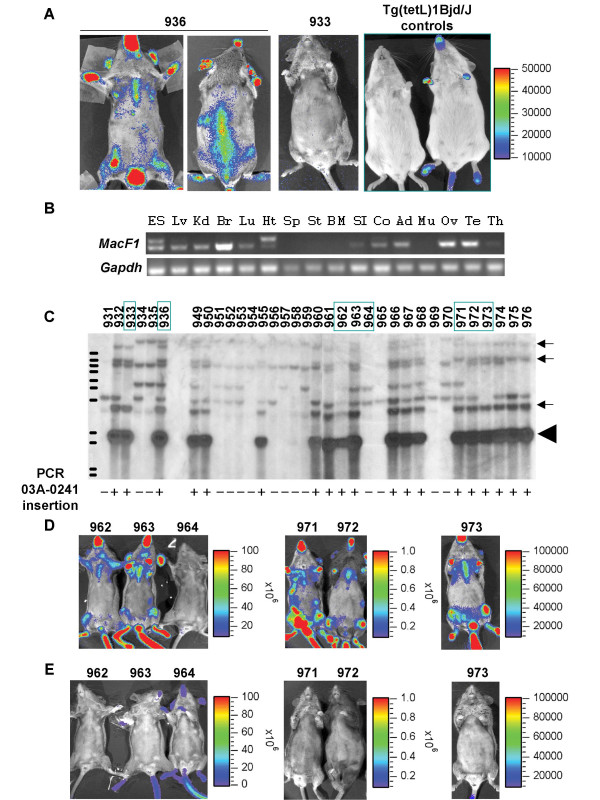
***In vivo *imaging of gene-trap tTAactivation of a tet-responsive transgene A)**. Luciferase emission pattern seen in a 03A-0241; Tg(tetL)1Bjd/J doubly transgenic mouse (936) as imaged by an intensified charge-coupled device (CCD) camera. Ventral and dorsal aspects are shown. The intensity of luciferase expression is compared in photons/second/cm^2^. **B) **RT-PCR analysis of *MacF1 *expression in multiple adult tissues, *Gapdh *was used as a control for sample quality. **ES**-embryonic stem cells, **Lv**-liver, **Kd**-kidney, **Br**-brain, **Lu**-lung, **Ht**-heart, **Sp**-spleen, **St**-stomach, **BM**-bone marrow, **SI**-small intestine, **Co**-colon, **Ad**-adipose, **Mu**-muscle, **Ov**-ovary, b-testes, **Th**-thymus **C) **Southern analysis of mice from **A **and **B **(see Materials and Methods). The donor site concatemer appears as an intense *Bam*HI fragment at 2236 base pairs (arrowhead). The detection of insertion 03A-0241 by three-primer PCR is shown below each lane. Three bands marked by arrows segregate with insertion 03A-0241 genotyping and the concatemer.**D) **Pattern inheritance by offspring of mouse 936. Mice were imaged and scaled to different ranges of intensity that ranged from 1x10^4 ^to 1×10^8 ^photons/second/cm^2 ^(scale bars). **E) **Repression of *in vivo *luciferase activation after six days of treatment with doxycycline in the same mice from **D**.

Hasan, et al. (2001) demonstrated that non-invasive imaging of luciferase expression of a similar tTA-activated strain using an intensified charge-coupled device camera system after subcutaneous injection of luciferin substrate [25]. We used the Xenogen IVIS™100 Imaging System to see if tissue-specific patterns could be detected in gene-trap tTA; Tg(tetL)1Bjd/J doubly transgenic mice. Light emissions are often observed in the extremities, nose, ears, and tails of control Tg(tetL)1Bjd/J in a tTA-independent manner in our hands (Fig. 5A). No additional tissue-specific signals were initially detected in 02A-0001; Tg(tetL)1Bjd/J or 02A-0002; Tg(tetL)1Bjd/J doubly transgenic mice, suggesting that expression of luciferase levels detected in the organ extracts were not sufficient to be detected by the imager. Likewise, when crossed to the Tg(tetL)1Bjd/J strain, insertions 03A-0184 and 03A-0217 (Fig. 4A) showed no activation of luciferase by imaging or by luminometer readings of organ extracts (data not shown). While the expression pattern of Membrane-spanning 4-domains, subfamily A, member 6C (*Ms4a6c*) is not characterized, Integrin beta 3 (*Itgb3*) has been localized to the developing mouse heart in early embryonic stages [26]. It is possible that we missed the expression of these gene-trap tTA insertions because these genes are not expressed in adult tissues.

In contrast, a striking pattern of luciferase expression was captured by imaging 03A-0241; Tg(tetL)1Bjd/J doubly transgenic mice (mouse 936) when compared to singly 03A-0241 transgenic (mouse 933) or Tg(tetL)1Bjd/J controls (Fig. 5A). Light emission patterns were detected from anatomical origins corresponding to the sternum, spleen, femurs and vertebrae, presumably reflecting luciferase activation in a component of the bone marrow and hematopoietic cells. The *MacF1 *gene has more than 100 exons and has been reported to be expressed in multiple isoforms in the mouse with high levels produced in the lung [27]. Using primers in exons that flank the 03A-0241 insertion to amplify *MacF1 *transcripts, Fig. 5B demonstrates that at least two splice variants of different sizes are variably expressed in several tissues, but *MacF1 *is not normally expressed in the bone marrow of a wild-type mouse. This suggests that the 03A-0241 insertion into *MacF1 *may cause abnormal expression of the gene in the bone marrow and hematopoietic cells. However, multiple attempts to amplify the chimeric *MacF1*-IRES-tTA transcript from bone marrow extracts from carrier mice by RT-PCR were unsuccessful in identifying this spliced transcript.

To determine whether this pattern was linked only to insertion 03A-0241 and the mouse *MacF1 *gene, Southern blots and genotyping PCR were performed on mouse 936 (and siblings 931–935) and his offspring (mice 949–976) (Fig. 5C). Surprisingly, mice that inherited insertion 03A-0241 by PCR showed linkage to the concatemer donor site by Southern blot (Fig. 5A, arrowhead). This was unexpected because these mice were generated from T2/GT3/tTA line 6660 (Table 1), which has a transposon donor site on mouse chromosome 11. *MacF1 *is located on mouse chromosome 4 at 57.4 cM, thus the Southern data suggests that a large portion of the chromosome-11 concatemer had translocated by some mechanism to chromosome-4. Thus, the *MacF1 *insertion on chromosome 4 is still genetically linked to a translocated donor concatemer of multiple T2/GT3/tTA transposons in addition to other linked single-copy insertions (Fig. 5C, arrows). Repeated attempts to identify these other linked chromosome-4 insertions by our cloning techniques and 5'RACE for trapped sequences in bone marrow extracts were unsuccessful in identifying the origin of this tissue-specific gene-trap tTA expression. Nevertheless, the pattern seen in 03A-0241; Tg(tetL)1Bjd/J mice is unique to these mice, as it is not seen in carriers of other T2/GT2/tTA or T2/GT3/tTA insertions (with or without associated concatemers) when crossed to the Tg(tetL)1Bjd/J strain (above and data not shown).

The tissue-specific luciferase pattern in carriers of this insertion could be reproducibly transmitted through the germline to offspring (mice 962–964 and 971–973, Fig. 5D). The upper range of luciferase activation from each offsrpring, measured in photons/second/cm^2^, ranged approximately a thousand-fold (scale bars). We attribute these differences to differences in the levels of cap-independent translation of the tTA molecule from the IRES in each mouse. Finally, other groups have demonstrated *in vivo *sensitivity of tTA-dependent activation of gene expression to tetracycline, or its analog doxycycline [24,28,29]. We determined whether we could control gene-trap tTA activation of luciferase by administering doxycycline to the drinking water of the 03A-0241; Tg(tetL)1Bjd/J mice. Fig. 5E demonstrates absence of luciferase signal in the same animals from Fig. 5D, after six days of treatment, suggesting complete repression of the tTA-dependent luciferase activation.

## Discussion

We examined the utility of a SB transposon-base gene trap vector that expresses the Tet-off transcriptional transactivator in the patterns of trapped genes *in vitro *and *in vivo*. The gene-trap tTA demonstrated full utility in cultured cells, having the ability to insert in genes, trap the splicing of those genes to express the tTA transcription factor which, in turn, could activate a TRE-regulated transgene in a doxycycline-dependent manner. Likewise, analysis of gene-trap tTA transposon insertions into mouse genes revealed that the vector system can completely disrupt the expression of a mouse gene in some cases and can tissue-specifically activate expression of a TRE promoter-regulated transgene in a doxycycline-dependent manner *in vivo*. While ultimately this vector system can function as designed, several observations suggest we need a more optimal approach for creating a resource of mice that express tTA in developmentally-controlled patterns.

Germline SB transposon mobilization rates in the mouse germline are variable and ranged from less than one in twenty to nearly three events per gamete. An evaluation of several different transposon-transgenic lines has given us insight into the potential the SB system to perform regional mutagenesis throughout the genome. The genomic location of the transposon donor site, along with copy number, impacts the germline mobilization rate from any concatemer (see Table 1). This suggests that not all parts of the genome are equally accessible to SB transposition. Understanding the mechanisms behind this "mobilization position effect" will likely require an increased understanding about molecular regulators of *SB *transposition, but may include a well known director of position effect, heterochromatinization [30].

As demonstrated in previous studies and here, *SB *transposon-based gene trap vectors can function *in vivo *to disrupt the expression of mouse genes. The T2/GT2/tTA vector can completely ablate the expression of a mouse gene as demonstrated by the null mutation that was generated in the mouse *Car12 *gene. Not all gene insertions completely disrupt gene expression however, as in the case of insertion 02A-0002 into *ENSMUSG00000066992*. While hypomorphic mutations may be valuable to discover the function of an essential mouse gene, this has lead to ideas on how to improve the efficacy of future vectors. Splice acceptors derived from different genes, termination sequences (polyadenylation signals) and other functional sequences have been used in several ES cell plasmid- and retroviral-based gene-trapping studies with varying efficiencies [31-33]. Drawing data from other groups will lead to improved vectors for trapping mouse genes and perhaps lead to vectors to trap certain classes of genes [34].

The activation of luciferase by the tetracycline-controlled transactivator varied rougly 1000-fold (Fig. 5D) when expressed from the IRES in the gene-trap tTA. Once again, improvements to the functional components of the gene trap vector may lead to more reliable and greater expression of the transactivator. Alternative IRES elements like the 9-nucleotide IRES element from the murine *Gtx *homeodomain gene [35], or eliminating the IRES entirely, may allow for more consistent tTA expression.

The brilliant pattern of *in vivo *luciferase expression seen if Fig. 5 was initially thought to originate from insertion in the *MacF1 *gene. Published reports suggested that neither the bone marrow, nor hematopoietic cells are in the normal expression domain of this gene. Upon further molecular characterization, we discovered that this gene was not spliced into the gene-trap tTA, and by Southern blot, found it was tightly linked to other insertions as well as the concatemer. It seems more likely that the pattern of tTA-dependent luciferase activation seen in carriers of the 03A-0241 insertion comes from one of these linked transposons. Efforts to find the source of this expression by 5'RACE were unfortunately unsuccessful. We propose that in the case of insertion 03A-0241, the concatemer translocated to chromosome 4 first, then a single copy of T2/GT3/tTA hopped locally into *MacF1*. We have observed similar and other types of genomic rearrangements associated with transposition from the chromosome-11 donor site in other mice (A.M.G., manuscript in preparation). These observations would suggest that gene-trap tTA mobilization in the germline may not be the most efficient means of generating a resource of mice with tissue-specific tTA expression patterns. It is not clear, however, that these large donor site insertions are not rare events that could be tolerated. Independent insertions, unlinked to the concatemer or other transposons, are frequently recovered. By pre-selecting mice that do not inherit the concatemer by Southern blot or PCR analysis, a screen would largely avoid the complications of linked transposons.

An alternative and relatively facile approach would be to inject a linearized plasmid harboring the gene-trap tTA transposon, along with *in vitro*-transcribed transposase messenger RNA, into one-cell mouse embryos. In the one-cell embryo, the transposase mRNA is translated and transposase catalyzes integration into the mouse genome at rates that are two to three-fold higher than standard pronuclear injection of naked DNA [3]. This method produces transgenic mice with insertions distributed randomly across the genome, and multiple linkage groups are frequently obtained from a single injection [3]. This would be another method to avoid the complications of linked insertions and concatemers.

Finally, using the *GAL4*/UAS enhancer trap systems used in the fly, expression-based screening has been useful for identifying genes with similar or overlapping expression patterns [36] and for tissue-specific overexpression studies [37]. As we have shown here, a transgenic mouse expressing firefly luciferase under the control of the tet-response enhancer/promoter (TRE) can be coupled with the gene-trap tTA system to identify *in vivo *patterns with an intensified CCD camera system. In a screen, this type of imaging could be used to identify gene trap insertions that lead to tTA expression in a particular tissue or developmental stage, even *in utero *[38,39]. Alternatively, mouse strains for other TRE-regulated transgenes have been created, allowing for tTA-dependent expression of β-galactosidase [40] or GFP [41]. If the efficiency of the gene-trap tTA transposon can be improved, these strains could be used by any lab to identify tissue-specific patterns.

## Conclusion

As a tool for the mouse community, introducing a gene-trap tTA vector into mouse genes with different spatial and temporal expression patterns would create a valuable tool for the mouse genetic toolbox. Tissue-specific promoters can control the expression of transactivators like tTA and rtTA (reverse tetracycline-controlled transactivator) in transgenic animals [42-44]. Having tissue-specific and temporal control of gene expression in any mouse tissue at any developmental stage could allow for the dissection of biological gene functions that were previously masked by a phenotype such as lethality. An SB-based transposon vector is well suited to accomplish this task by introducing precisely integrated functional reporter units into the mouse genome. In addition, an improved gene-trap tTA could provide more reliable expression of the transactivator than a transgene construct since transcription is regulated in the context of an endogenous gene and is thus less likely to be subject to the position effect variegation that can hinder the expression of transgenes.

## Methods

### Cloning gene-trap tTA transposon vectors

All primer/oligo sequences for PCR and cloning are provided in Additional file 4. All gene-trap tTA vectors are descendent of pGT/tTA, originally described in Clark *et al *. 2004 [13]. An artificial exon (based on carp β-actin exon 2), with stop codons in each reading frame, was created by annealing four oligos (AMG049-AMG052), filling in the gaps with Klenow DNA polymerase, ligation with T4 DNA ligase, PCR amplifying with AMG049 and AMG051, and subcloning the *Age*I fragment into the partially-digested *Sma*I-*Age*I fragment of pGT/tTA to create pGT2/tTA. Subcloning the exonuclease-treated *Eco*RV-*Pst*I fragment of pGT2/tTA into the Klenow-treated blunt *Hind*III fragment of pT2/HindIII [14] generated pT2/GT2/tTA. pT2/GT2/SVNeo was created by subcloning the Klenow-treated *Hind*III fragment of pT2/SVneo [14] into the *Eco*RV fragment of pT2/GT2/tTA.

The construction of pT2/GT3/tTA was a multi-step process to add additional features to the GT2 version. First, an *Nhe*I site was added to pT2/GT2/tTA by PCR amplifying the entire plasmid with primers AG023 and AG024, containing a 12-bp overlap containing the *Nhe*I site sequence, and transformation. *E. coli *repairs the plasmid by homologous recombination in the overlapped region, resulting in the plasmid pT2/GT2/tTA/NheI. A loxP site was made by annealing two oligos (AG025 and AG026) containing the 34-bp loxP sequence with overhanging 4-base cohesive ends to *Nhe*I and cloned into the *Nhe*I site of pT2/GT2/tTA/NheI, destroying the *Nhe*I site on one side, and adding the loxP sequence to make pT2/GT2/tTA/loxP. The *Nhe*I-*Spe*I fragment of pT2/GT2/tTA/loxP was subcloned into the *Xba*I fragment of the transposon vector pT2/HB, which has a multiple cloning site, to create pT2/GT2/tTA/loxP-2. 70-mer oligos (SA/V5-1 and SA/V5-2) containing the HPRT splice acceptor and V5 epitope tag with *Eag*I compatible ends were annealed and cloned into the *Eag*I site of pT2/GT2/tTA/loxP-2 to make pT2/GT2/tTA/loxp/SA-V5. In the process, one side of the *Eag*I site was destroyed, leaving a single *Eag*I site. A 32-bp transcriptional termination sequence from the human *GASTRIN *gene was created by overlapping oligos (Termin-1 and Termin-2) and cloned into this *Eag*I site of pT2/GT2/tTA/loxp/SA-V5 to make pT2/GT2/tTA/loxp/SA-V5/Termin. This sequence was shown to enhance transcriptional termination in plasmid vectors when downstream of polyadenylation signals [45], and was included to increase the chances of mRNA truncation, and thus mutation, in the gene trap. Finally, a second loxP site was made by annealing the same oligos above and ligation into the *Spe*I site of pT2/GT2/tTA/loxp/SA-V5/Termin to make the final product, pT2/GT3/tTA.

### Generation of Tre-Luciferase/Puro cell line and in vitro gene trap testing

pTRE-Luc was kindly provided by Dr. Perry Hackett's lab and was co-transfected with the plasmid pKO Select-Puro (Stratagene, La Jolla, CA) into HeLa cells using Mirus TransIT-LT1 reagent (Promega, Madison, WI). After puromycin resistant clones were isolated, ten clones were tested for inducibility by pTet-off (Clontech, Palo Alto, CA). One clone TLP-10 showed a four-log increase in luciferase expression over background in the presence of transfected pTet-off (data not shown), and was subsequently used for the *in vitro *studies presented here.

pT2/GT2/tTA/SVNeo was transfected into the TLP-10 cell line along with pCMV-SB [1], and clones were selected in 800 μg/mL G418. pT2/SVNeo [14] was used as a control. Fifty of these clones and twelve control clones were grown to confluency on 60 mm plates and cells harvested with Promega's Cell Culture Lysis Reagent for luciferase assays. Luciferase assays were performed on 20 μL of lysis extract using 100 μL of Promega's Luciferase Assay Substrate and a Lumat LB 9507 luminometer (Berthold, Bundoora, Australia) with a 15 second measuring time. Relative light unit (RLU) measurements were normalized to total protein concentration as determined by Bradford assay. Doxycycline (Sigma cat. #D-9891) was added to a final concentration of 2 mM to repress tTA-induction of TRE-Luciferase in clones 39, 43, and 44.

### RT-PCR and 5' rapid amplification of cDNA ends (RACE)

All primer/oligo sequences for PCR are provided in Additional file 4. Total RNA from cultured cells or mouse tissues was extracted with Trizol^® ^(Invitrogen, Carlsbad, CA). RT-PCR was performed using the one-step RobusT™ I RT-PCR Kit (Finnzymes, Espoo, Finland) according to the manufacturer's protocol. A template-switching reaction with Superscript III (Invitrogen) was used to make template for 5'RACE on cultured cell or mouse tissue RNA extract. 250 ng total RNA was mixed with 1 μM each of primers GT2-RT3 and RACE-1, along with 0.01 M dithiothreitol, 500 μM dNTPs, and 1 unit of Superscript III. The mixture was cycled six times at 52°C for 10', 50°C for 15', and then gradually cooled from 50°C to 37°C over two minutes before a final extension for 90" at 37°C. Template was diluted ten-fold in water and 2 μL was used for primary 5'RACE PCR. Briefly, the template was amplified in a 50 μL PCR reaction supplemented with primers GT2-RT3 (0.4 μM), KJC-002 (0.04 μM) and KJC-003 (0.1 μM), 200 μM dNTPS, 2 mM MgCl_2_, and 1 unit of Taq DNA polymerase (CLP, San Diego, CA). The PCR machine was programmed for touchdown PCR at 95°C for 3', 10 cycles of 95°C for 30", 65°C for 30" (-0.5°C per cycle), 70°C for 2', and then 25 cycles of 95°C for 30", 60°C for 30", and 70°C for 2'. The primary RACE reaction was diluted 1:50 and 2 μL used in a nested PCR under the same exact conditions, except supplemented with 0.5 μM of each primer GT2-RT2 and KJC-004.

### Generation of T2/GT2/tTA and T2/GT3/tTA mice by pronuclear injection, fluorescent in situ hybridization, and Southern blotting

The 3379-bp *Fsp*I *-Sap*I fragment of pT2/GT2/tTA or the 3738-bp *Fsp*I *-Sap*I fragment of pT2/GT3/tTA were gel-purified, ethanol precipitated twice, and resuspended in 5 mM Tris-Cl (pH 7.5), 0.1 mM EDTA for pronuclear injection into the FVB/N strain of mice (Charles River Laboratories, Wilmington, MA). The pT2/GT3/tTA fragment was co-injected with the similarly-prepared *HinP1*I fragment of the plasmid pTYBS [Overbeek, 1991 #211], a tyrosinase minigene construct kindly provided by Dr. Paul Overbeek, in an attempt to coat-color mark animals that inherit the transposon/tyrosinase minigene transgenic insertion site. Expression of the tyrosinase, for unknown reasons, was not evident in any of the founders (data not shown). FISH on T2/GT3/tTA transgenic mouse lines was performed on splenic lymphocytes using standard techniques, and performing nick translation to label the pT2/GT3/tTA plasmid or whole BAC DNA from the Wellcome Trust Sanger Institute [46] were used as a probe. Standard Southern blotting techniques were used to analyze *Bam*HI-digested genomic DNA from founders and subsequent generations using the 842-bp *Nco*I *-Sph*I fragment of the tTA open reading frame as a probe. *Bam*HI restricts each copy in any T2/GT2/tTA or T2/GT3/tTA concatemer to a single band at 2124-bp or 2236-bp respectively (see Fig. 1A). Transposons mobilized from the concatemer to a genomic site are detected as bands of variable size which are larger than the concatemer band, their size determined by the nearest genomic *Bam*HI site (see Fig. 5C).

### Western blotting for CA-XII expression

Whole kidney extracts were probed with a polyclonal CA-XII antibody kindly provided by the laboratory of Dr. William Sly. 50 μg total protein was run on a 10% SDS-PAGE (Invitrogen) and transferred to PVDF and probed with a 1:1000 dilution of anti-CAXII primary antibody followed by a 1:10000 dilution of horseradish peroxidase-conjugated Anti-rabbit IgG secondary (Amersham Biosciences) in TBST (10 mM Tris-HCl, pH 7.5, 150 mM NaCl, 0.1 % Tween 20) supplemented with 1% dry milk.

### *In vivo *luciferase imaging and analysis of luciferase expression, doxycycline treatment

Transgenic mice were imaged as described in Wilber, *et al *. (2005) [47], using luciferin from Xenogen Corporation (Alameda, CA). For luciferase assays on organ extracts, mice were sacrificed, and whole organs dissected and homogenized in Promega's Cell Culture Lysis Reagent. Extracts were analyzed as described above for the *in vitro *work. Drinking water was supplemented with 5 mg/mL Doxycycline and 0.1% sodium saccharine for 6 days to suppress tTA-induced activation.

## Authors' contributions

AMG performed the updated vector construction, *in vitro *testing of the gene-trap tTA, preparation of transgenes, breeding and characterization of transgenic mice, 5'RACE, RT-PCR, western blotting, Southern blotting, and aided the *in vivo *imaging studies. AW performed the *in vivo *imaging for luciferase expression. CMC bred and characterized 'rescue by remobilization' for insertion 01-0032. PDL performed PCR-based methods to identify gene-trap tTA insertions in transgenic mice. KJC originally designed the gene-trap tTA system and was instrumental to upgrading its design and functionality. PBH, RSM, and DAL are not only the mentors of the above authors, but were involved in the design and execution of this manuscript.

## Supplementary Material

Additional File 1 **T2/GT2/tTA/SVNeo clone 5'RACE sequence and gene identification **Splicing events of genes into the gene-trap tTA were identified by 5'RACE PCR and sequencing. For seven clones, the partial sequence tag demonstrates splicing into the proper branch point of the carp β-actin splice acceptor, allowing identification of the trapped gene by the sequence of the adjoined endogenous exon sequence. These genes and their functions are described according to the ENSEMBL May 17, 2005 freeze of the NCBI m34 build. Click here for file

Additional File 2 **Germline T2/GT2/tTA insertions **The genomic positions of thirty T2/GT2/tTA insertions identified in the offspring of seed mice from line 4563 (Table 1) are reported. Shown are the chromosome and position, along with the identification and description of known or predicted genes potentially disrupted for each insertion. This data is based on the ENSEMBL May17, 2005 freeze of the NCBI m34 build. Click here for file

Additional File 3 **Distribution of thirty T2/GT2/tTA insertions **Cloning insertions from offspring of seed mice from transgenic line 4563 (Table 1) reveals two local hopping intervals, one on mouse chromosome 1 near 45.8 Mb, and a second on mouse chromosome-9 around 66.5 Mb. By Southern blot, it was later determined that the 4563 line of mice originally obtained and segregated two independent concatemer integrations during the initial transgenesis (data not shown) Click here for file

Additional File 4 **Primer sequences** Click here for file

## Figures and Tables

**Table 1:** Mobilization of gene-trap tTA transposons in the germlines of seed mice.

Vector	**Line**	**Chr. (Pos)**	**Copy No. ^*a*^**	**Seed Mice (T2+/-;SB10+/-)**	**Offspring**	**New Hits**^*b*^	**Hits/Gamete**	**Rate/Copy No.**^*c*^
	4555	ND	3	**M**	10	0	<1:10	**NA**
				**F**	13	0	<1:13	
				**M**	10	14	1.40	
				**M**	19	23	1.21	
	4563	ND	50	**F**	10	6	0.60	**0.019**
				**M (SB10+/+)**	10	12	1.20	
				**M**	11	3	0.27	
T2/GT2/tTA				**M**	33	24	0.73	
				**M**	12	10	0.83	
				**M**	8	4	0.50	
				**M**	19	10	0.53	
	4583	ND	60	**F**	8	1	0.13	**0.008**
				**M**	17	13	0.76	
				**F (pT2 +/+)**	8	1	0.13	
				**M**	10	3	0.30	
	4587	ND	10	**M**	21	0	<1:21	**NA**

	6630	ND	4	**M**	20	1	0.05	**0.020**
				**M**	18	2	0.11	
	6632	**6 (B3-C1)**	5	**M**	15	20	1.33	**0.267**
	6657	**7 (A3-B2)**	10	**M**	18	8	0.44	**0.044**
				**F**	10	10	1.00	
T2/GT3/tTA	6660	**11 (B5-C)**	27	**M**	7	26	3.71	**0.098**
				**M**	10	18	1.80	
				**M**	16	43	2.69	
				**M**	26	79	3.04	
				**M**	16	59	3.69	
	6682	**3 (E1-E3)** **10 (C3-D1)**	11	**M**	16	6	0.38	**0.063**
	6683	**9 (F1-F2)**	6	**M**	14	14	1.00	**0.091**

^*a *^Transposon copy number for each transgenic line was determined by Southern blot (see Materials and Methods)

^*b *^The number of single-copy insertions as determined by the presence of new bands on a Southern blot after mobilization by transposase in the germline of seed mice

^*c *^Reported is the average hits/gemete divided by the copy number
