# Supplementary figures and images for: Conditional gene expression in the mouse using a Sleeping Beauty gene-trap transposon
Source: BMC Biotechnol. 2006 Jun 26;6:30. doi: 10.1186/1472-6750-6-30 (PMC1557845; doi:10.1186/1472-6750-6-30)

**Additional file 3 – Distribution of thirty T2/GT2/tTA insertions**

**
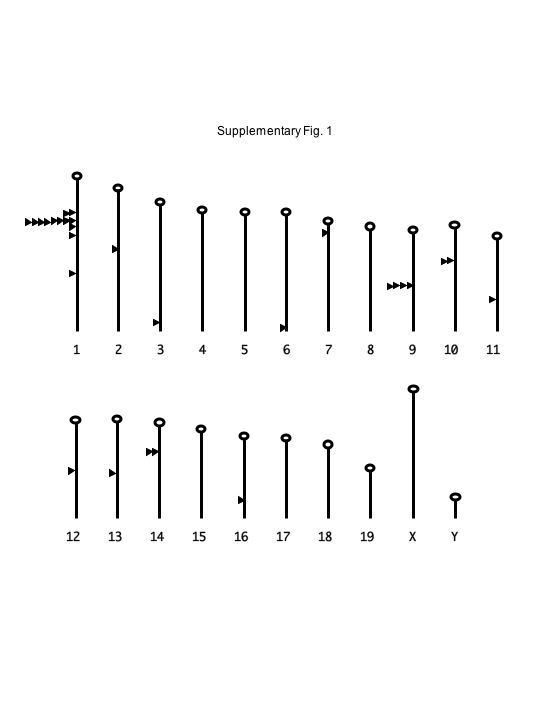
**

Supplement: Additional File 3 — Distribution of thirty T2/GT2/tTA insertions Cloning insertions from offspring of seed mice from transgenic line 4563 (Table 1) reveals two local hopping intervals, one on mouse chromosome 1 near 45.8 Mb, and a second on mouse chromosome-9 around 66.5 Mb. By Southern blot, it was later determined that the 4563 line of mice originally obtained and segregated two independent concatemer integrations during the initial transgenesis (data not shown) [file 1472-6750-6-30-S3.doc]
