# Supplementary material for: Conditional gene expression in the mouse using a Sleeping Beauty gene-trap transposon
Source: BMC Biotechnol. 2006 Jun 26;6:30. doi: 10.1186/1472-6750-6-30 (PMC1557845; doi:10.1186/1472-6750-6-30)
Supplement: Supplementary file 2 [file 1472-6750-6-30-S1.doc]

Additional file 1: T2/GT2/tTA/SVNeo clone 5'RACE sequence and gene identification

Clone #	Partial Sequence tag	Chr.	Gene ID	Function (ENSEMBL database)
15	GCCCTGAACACGGGCAAGCTGCAGCAGCAGCCTGGCTGCACTGCTGAGGAGACCCTGGAG***TTCAGCCGATGATGAAATTGCCGCACTGGT***	10	RPC1	DNA-DIRECTED RNA POLYMERASE III LARGEST SUBUNIT
33	GAACTACAACAAGAAGCAGCTCTTATTAGAAGTCGAGAAAAACCGTTGGCTCTGATTCAG***TTCAGCCGATGATGAAATTGCCGCACTGGT***	6	Q9P2D0	No description
37	TATCTTGATATTCCATTGGTCATCCGACCTTATGGGTCCAGCCAAGCATTTGCTAGTGTG***TTCAGCCGATGATGAAATTGCCGCACTGGT***	11	USP47	ubiquitin specific protease 47
43	AAACTATCGCTGAGGACCTGGTCGTGACCAAGTATAAGATGGGGGGCGACATCGCCAACA***TTCAGCCGATGATGAAATTGCCGCACTGGT***	12	PA2G4	PROLIFERATION-ASSOCIATED PROTEIN 2G4
44	TTCGCCTTCGTGGAGTTTTATCACTTGCAAGATGCTACCAGCTGGATGGAAGCCAATCAG***TTCAGCCGATGATGAAATTGCCGCACTGGT***	3	RBM5	RNA-BINDING PROTEIN 5
50	CAGAAGATCCTTCTGTAGTATATTTTCAGTGCTGCCGTATCGCGACAGTACCCAAGTCGG***TTCAGCCGATGATGAAATTGCCGCACTGGT***	18	CABLES1	Cdk5 and Abl enzyme substrate 1
55	GGGCTGGGAAGGTGCCAAGTACGCAGTGGCCGTGGGCTCCCTCTGCGCTCTTTCCGCCAG***TTCAGCCGATGATGAAATTGCCGCACTGGT***	13	SLC7A1	HIGH-AFFINITY CATIONIC AMINO ACID TRANSPORTER-1 (CAT-1)
