# Supplementary material for: Conditional gene expression in the mouse using a Sleeping Beauty gene-trap transposon
Source: BMC Biotechnol. 2006 Jun 26;6:30. doi: 10.1186/1472-6750-6-30 (PMC1557845; doi:10.1186/1472-6750-6-30)
Supplement: Supplementary file 3 [file 1472-6750-6-30-S2.doc]

Additional file 2: Germline T2/GT2/tTA insertions

ID	Chr.	Position	gene, genescan, or expressed sequence tag-hit	Description
02A-0001	9	66,841,111	Car12 (+)	Carbonic anhydrase XII precursor
02A-0002	1	116,569,669	ENSMUSG00000066992	no description
02A-0003	12	57,890,743	nothing	
02A-0004	1	33,346,457	nothing	
02A-0005	1	35,905,988	nothing	
02A-0006	1	44,508,925	ENSMUSG00000046828 (Q8CD72) (+)	no description
02A-0007	1	45,609,982	nothing	
02A-0008	1	44,969,056	Gulp1 (+), GENSCAN (-)	GULP, engulfment adaptor PTB domain containing 1 [Source:MarkerSymbol;Acc:MGI:1920407]
02A-0009	1	47,241,100	ENSMUSG00000067518 (+)	no description
02A-0010	1	46,343,131	ENSMUSG00000067542 (-)	no description
02A-0011	1	44,931,588	Gulp1 (-), GENSCAN (+)	GULP, engulfment adaptor PTB domain containing 1 [Source:MarkerSymbol;Acc:MGI:1920407]
02A-0012	1	46,529,551	ENSMUSG00000067536 (-)	PREDICTED: similar to axonemal dynein heavy chain 7 Source: RefSeq_peptide_predicted XP_484861
02A-0013	1	46,180,219	ENSMUSG00000067542 (-)	
02A-0014	1	55,567,242	XP_283621.3	PREDICTED: similar to axonemal dynein heavy chain 7 Source: RefSeq_peptide_predicted XP_283621
02A-0015	1	65,585,628	nothing	
02A-0016	13	60,041,247	ENSMUSG00000058403 (+)	no description
02A-0017	14	34,205,367	nothing	
02A-0018	14	34,233,409	nothing	
02A-0019	2	73,446,204	Chn1 (+), GENSCAN (-)	chimerin (chimaerin) 1 [Source:MarkerSymbol;Acc:MGI:1915674]
02A-0020	9	65,458,832	NP_001028330.1 (+), GENSCAN (-)	RAS-like, family 12 Source: RefSeq_peptide NP_001028330
02A-0021	9	66,373,940	Dapk2 (-)	death-associated kinase 2 [Source:MarkerSymbol;Acc:MGI:1341297]
02A-0022	9	67,584,258	ENSMUSESTG (+)	no description
02A-0023	10	89,659,773	nothing	
02A-0024	10	89,538,960	nothing	
02A-0025	11	78,578,598	GENSCAN (-)	no description
02A-0026	16	76,737,549	GENESCAN (+), ENSMUSESTT (+)	no description
02A-0027	6	142,911,622	nothing	
02A-0028	7	7,735,030	ENSMUSG00000066828 (-)	no description
02A-0029	MANY		L1 element	
02A-0030	3	145,485,283	Bxdc5 (-)	brix domain containing 5 [Source:MarkerSymbol;Acc:MGI:1917535]
