# Supplementary material for: Conditional gene expression in the mouse using a Sleeping Beauty gene-trap transposon
Source: BMC Biotechnol. 2006 Jun 26;6:30. doi: 10.1186/1472-6750-6-30 (PMC1557845; doi:10.1186/1472-6750-6-30)
Supplement: Supplementary file 4 [file 1472-6750-6-30-S4.doc]

Additional file 4: Primer sequences

Primer	Sequence (5' to 3')
GT2-RT2	CTAACAACCAGTGCGGCAATTT
GT2-RT3	CCGGCTACGTTGCTAACAACC
RACE-I	CCAAGTTCGTACAAAAAAGCAGGCTCGGGG
KJC-002	AATGGCTTCCGAGACCTGCTGTCCAAGTTCGTACAAAAAAGCAGGC
KJC-003	AATGGCTTCCGAGACCTGCTGT
KCJ-004	GAGACCTGCTGTCCAAGTTCGT
AMG-049	TTCTAAACCGGTTTTCTCATTCATTTACAGTTCAGC
AMG-050	TGCCGCACTGGTTGTTAGCAACGTAGCCGGTATGT
AMG-051	GCGAATCCATCTTTCACATACCGGCTAC
AMG-052	CCAGTGCGGCAATTTCATCATCGGCTGAACTGTAAATG
AG023	GGCAATGCTAGCAAATACTAATTGAGTG
AG024	GCTAGCATTGCCTTTAAATTGTTTAACTT
AG025	CTAGCATAACTTCGTATAGCATACATTATACGAAGTTAT
AG026	CTAGATAACTTCGTATAATGTATGCTATACGAAGTTATG
SA/V5-1	GGCCTGATTTTATTTCTATAGGACGGTAAGCCTATCCCTAACCCTCTCCTCGGTCTCGATTCTACGTGAC
SA/V5-2	GGCCGTCACGTAGAATCGAGACCGAGGAGAGGGTTAGGGATAGGCTTACCGTCCTATAGAAATAAAATCA
TERMIN-1	GGCCAAAAAAAAATTAAAAATAAAATAAAATAAAAA
TERMIN-2	GGCCTTTTTATTTTATTTTATTTTTAATTTTTTTTT
**Genotyping primers (for three-primer insert or simple transgene genotyping)**	
**Inserts**	
Trap geno	GCTACTCGAAATGTTTGACCCAAG
02A-0001A	ATTCTAGGAAGAGCTTGACTCCCAC
02A-0001B	ATTCTCATCTGAGTTTCAATGCCAG
02A-0002A	AGTCTCCTGAGAAACTCCTCAATCTACT
02A-0002B	CAAGAGAGCTTAGTTGGTACAGTGATTT
03A-0184A	CAACAGATGTAGAGATGCACCATGG
03A-0184B	GGGCTTCAGAGTTTTGTGATTCCT
03A-0217A	TTTTTCAGTGGACTCTCAAATCACG
03A-0217B	CCTTCCTCTCCTTTAGCAGTAAACTAAGA
03A-0241A	CTTACTTTCTTTCTCATTAGAGTGAGTCCG
03A-0241B	GGTCCTAAATTCAATTCCCAACCA
**Transgenes**	
Luc-1	TGCACATATCGAGGTGAACATCA
Luc-2	TCAAAGGACTCTGGTACAAAATCGT
